# Minimally Invasive Direct Coronary Artery Bypass Grafting: Sixteen Years of Single-Center Experience

**DOI:** 10.3390/jcm13113338

**Published:** 2024-06-05

**Authors:** Alexander Weymann, Lukman Amanov, Eleftherios Beltsios, Arian Arjomandi Rad, Marcin Szczechowicz, Ali Saad Merzah, Sadeq Ali-Hasan-Al-Saegh, Bastian Schmack, Issam Ismail, Aron-Frederik Popov, Arjang Ruhparwar, Alina Zubarevich

**Affiliations:** 1 Department of Cardiothoracic, Transplant and Vascular Surgery, Hannover Medical School, 30625 Hannover, Germanymerzah.ali@mh-hannover.de (A.S.M.);; 2 Medical Sciences Division, University of Oxford, Oxford OX1 2JD, UK; 3 Department of Cardiac Surgery, University Hospital Halle, 06120 Halle (Saale), Germany

**Keywords:** MIDCAB, coronary revascularization, minimally invasive surgery

## Abstract

**Background**: Coronary artery disease is a major cause of death globally. Minimally invasive direct coronary artery bypass (MIDCAB), using a small left anterior thoracotomy, aims to provide a less invasive alternative to traditional procedures, potentially improving patient outcomes with reduced recovery times. **Methods:** This retrospective, non-randomized study analyzed 310 patients who underwent MIDCAB between July 1999 and April 2022. Data were collected on demographics, clinical characteristics, operative and postoperative outcomes, and follow-up mortality and morbidity. Statistical analysis was conducted using IBM SPSS, with survival curves generated via the Kaplan–Meier method. **Results:** The cohort had a mean age of 63.3 ± 10.9 years, with 30.6% females. The majority of surgeries were elective (76.1%), with an average operating time of 129.7 ± 35.3 min. The median rate of intraoperative blood transfusions was 0.0 (CI 0.0–2.0) Units. The mean in-hospital stay was 8.7 ± 5.5 days, and the median ICU stay was just one day. Early postoperative complications were minimal, with a 0.64% in-hospital mortality rate. The 6-month and 1-year mortalities were 0.97%, with a 10-year survival rate of 94.3%. There were two cases of perioperative myocardial infarction and no instances of stroke or new onset dialysis. **Conclusions:** The MIDCAB approach demonstrates significant benefits in terms of patient recovery and long-term outcomes, offering a viable and effective alternative for patients suitable for less invasive procedures. Our results suggest that MIDCAB is a safe option with favorable survival rates, justifying its consideration in high-volume centers focused on minimally invasive techniques.

## 1. Introduction

Coronary artery disease stands as one of the leading causes of death in Western nations. The inception of coronary artery bypass grafting (CABG) in the 1960s marked its swift ascent to being amongst the most frequently conducted surgical interventions [1]. Over the years, significant improvements in outcomes have been observed, marked by reductions in both operative mortality and major morbidity rates [2]. Although outcomes have improved significantly in recent decades, the aging demographic of cardiac surgery patients, coupled with their increased comorbidities, has highlighted the necessity for less invasive surgical techniques [3]. Technological and engineering advancements over the last few decades have paved the way for the development of minimally invasive surgical methods, including the use of endoscopy in various procedures such as cardiac surgery [4]. Amongst the new perspectives in cardiac surgery, an alternative approach to a standard sternotomy for CABG has been implemented in recent years to facilitate a minimally invasive direct coronary artery bypass (MIDCAB) through a small left anterior thoracotomy. The left internal mammary artery (LIMA) can then be harvested either via direct vision or using endoscopic techniques [2]. Although the MIDCAB approach is commonly associated with increased postoperative pain for the patients, recovery appears to be faster than after a sternotomy, with potential improvement in the overall postoperative quality of life for the patients [2]. 

The MIDCAB approach may offer promising benefits for patients requiring surgical myocardial revascularization. However, its technically demanding nature and the lack of long-term outcomes remain major burdens for its further adoption [5]. The purpose of this retrospective study is to review and present the results of our single-center experience with MIDCAB for surgical myocardial revascularization.

## 2. Materials and Methods

### 2.1. Study Design 

This study is a single-centered, retrospective, nonrandomized study including 310 patients who underwent a MIDCAB operation between July 1999 and April 2022 at our institution.

### 2.2. Inclusion Criteria—Population

Every patient who underwent a MIDCAB operation at our institution between July 1999 and April 2022 was eligible for this study. Both elective, urgent, and emergent cases were included in the present study. The decision to perform a MIDCAB operation was individualized for each patient based on the coronary anatomy and was made at the surgeons’ discretion, always in accordance with the latest guidelines, our institutional multidisciplinary Heart Team decision, and patients’ informed consent.

### 2.3. Operative Technique

Under general anesthesia and with single-lumen intubation, the patient was placed on the operating table in a supine position with a slightly elevated left scapula. A small (5–8 cm) anterolateral thoracotomy was performed in the left fourth intercostal space. Transesophageal echocardiography and ECG were used to monitor ventricular function throughout the procedure. A specialized rib retractor (Thoralift^TM^ and, in the most recent cases, Mutistation^TM^, LSI Solutions, Victor, NY, USA) was selected to partially elevate the rib cage to achieve better visibility during LIMA harvesting. In order to achieve a sufficient length to reach the coronary artery without tension, the artery was mobilized to the highest possible level (up to the subclavian vein). Heparin was administered intravenously until the target PTT of >300 s had been reached. A stabilization device (Octopus^TM^, Medtronic, Minneapolis, MN, USA) was positioned to expose the LAD, and a longitudinal incision was made in the coronary artery. The LIMA graft was carefully prepared for the bypass. A shunt was placed into the coronary vessel and the end-to-side anastomosis was performed with 7/0 polypropylene (Prolene^TM^) suture in a running fashion. The shunt was removed, and the anastomosis was completed. The bulldog clamp was removed from the LIMA and the coronary flow was restored. Heparin was reversed and after completing the flow measurement, the wound was closed in anatomic layers. The patient was delivered intubated to the intensive care unit to be extubated in the following hours.

### 2.4. Data Acquisition 

In accordance with the data protection regulations, demographic information, clinical characteristics, and operative and postoperative data were retrospectively extracted from the institutional medical records of the included patients. Telephone interviews with the patients or/and their relatives or/and their primary care physicians were performed for an active follow-up. 

Due to the retrospective, observation nature of the study, the requirement for informed consent was deferred. This study was performed in accordance with the Declaration of Helsinki, and the data regarding the patient’s identity remained strictly anonymous. Ethical approval was obtained from the Ethics Committee of the Hannover Medical School, Hannover, Germany (Nr.11333_BO_K_2024, 4 April 2024). All methods utilized in the present study were performed in accordance with regulations and guidelines.

### 2.5. Definitions and Outcomes 

The primary endpoints of the present study were 30-day, 6-month, and 1-year mortality, as well as the overall survival at 5 and 10 years. The main secondary endpoint was the development of any postoperative adverse events. Data regarding the in-hospital stay, the intraoperative time, the need for transfusion, total ventilation time, conversion to sternotomy, and the need for surgical revision were also retrospectively retrieved. Urgent procedures were defined as procedures which had to be performed during the first 48 h after hospital admission. Emergent procedures were defined as procedures which had to be performed during the first 2 h after hospital admission.

Postoperative myocardial infarction was defined as a significant elevation of the CK-MB levels over 10% of the CK levels and a clinical correlate such as new changes in the ECG (ST-elevation or T-depression) and/or new dyskinesia detected by echocardiography. Hyperlipoproteinemia was defined as a state of abnormally high levels of triglycerides in blood (>150 mg/dL), requiring medical therapy. Kidney function impairment was defined as a reduction in GRF rate under 50 mL/min. 

### 2.6. Statistical Analysis 

The obtained data were entered into a dedicated Microsoft Excel spreadsheet. Statistical analysis was performed using IBM SPSS version 28 (IBM Corp., Chicago, IL, USA). Data were tested for normality using the Shapiro–Wilk test. When the data were not normally distributed, continuous variables were expressed as medians (interquartile range, IQR) or as mean ± standard deviation. Survival curves were generated using the Kaplan–Meier method. Categorical variables were expressed as frequencies and percentages. 

## 3. Results

### 3.1. Baseline Characteristics

The mean age of the patients was 63.3 ± 10.9 years, and 30.6% of the patients were female (Table 1). All patients presented with symptomatic coronary artery disease, with 5.4% of the patients (n = 17) suffering from acute myocardial infarction. Interestingly, the EuroSCORE II rates in those seventeen patients did not significantly differ from the rest of the cohort; therefore, there was no hesitation in performing the procedure via minimally invasive access. A large portion of the cohort (73 patients (23.5%)) had previously undergone a PCI, 30% of the patients were active smokers, and 57 patients (18.4%) were suffering from type 2 diabetes mellitus. The patients presented with a mean left ventricular ejection fraction of 57.1 ± 6.5% and a median EuroSCORE II of 0.9 (0.7–1.2), putting the patients into the low-risk group. Further details of patients’ baseline data are presented in Table 1.

### 3.2. Intraoperative Characteristics

In all cases, one anastomosis was performed (left internal mammary artery to ramus interventricularis anterior) on the beating heart without the cardiopulmonary bypass support. In 1% of the patients (n = 3), due to the problems with the anastomosis, the procedure had to be switched to a median sternotomy, and the anastomosis was performed on-pump with a beating heart. None of the patients had been considered for hybrid revascularization. 

The mean operating time was 129.7 ± 35.3 min, and in most cases, the median rate of intraoperative blood transfusions was 0.0 (CI 0.0–2.0) Units with a mean requirement of 0.35 Units per patient. The majority of the cases were elective (76.1%). Further intraoperative data are presented in Table 2. 

### 3.3. Overall Survival and Postoperative Outcomes

The mean in-hospital stay duration was 8.7 ± 5.5 days and the median ICU-stay duration was 1(0.-1.0) day. Two patients suffered from perioperative myocardial infarction (0.67%), and in those cases, coronary angiography was performed. There were no cases of stroke in our cohort, and in four cases (1.3%), re-exploration for bleeding was necessary. There was no relevant need for postoperative blood transfusion in our cohort (Table 3). 

**Figure 1:**
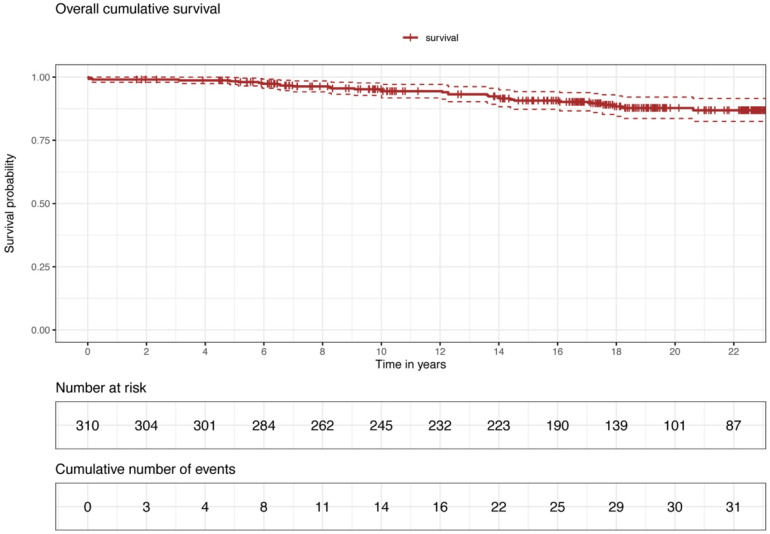
Overall survival.

The mean follow-up time was 16.3 ± 6.3 years. In-hospital mortality was 0.64% (n = 2), 6-month mortality was 0.97% (n = 3), and 1-year mortality was 0.97% (n = 3). We report a 5-year and 10-year mortality of 1.3% and 5.7%. Kaplan–Meier curve portrays the overall survival of our cohort (Figure 1).

The causes of death during the follow-up period are expressed in Table 4.

During the follow-up period, 63 patients (20.3%) underwent a diagnostic coronary angiography. In five patients (1.6%), LIMA-LAD bypass was not patent (after 4, 5 (×2), 7, and 18 years postoperatively).

## 4. Discussion 

In recent years, MIDCAB surgery presented promising results as an effective and safe minimally invasive approach for coronary revascularization [4,6,7]. The less invasive nature of this approach provides advantages for various patient groups, including those with comorbidities that make a standard sternotomy impractical [5,7]. The present single-center, retrospective study reports our results on long-term survival and postoperative results of consecutive patients who underwent MIDCAB revascularization in our institution between July 1999 and April 2022. Our results further support that MIDCAB revascularization is an effective and safe strategy. 

The mean operation time in our cohort was 129.7 ± 35.3, which is lower than the previously reported data [8,9]. The present study reports comparable short-term results with respect to the literature with an in-hospital mortality of 0.64%, a 6-month mortality of 0.97%, and a 1-year mortality of 0.97%. The mean follow-up time in the present study was 16.3 ± 6.3 years, which was longer than most of the comparable studies [7,8,9,10,11]. Our results regarding the 10-year survival rate (94.3%) for the entire cohort are favorable compared to the existing evidence in the literature [7,12,13,14].

Previously published studies reported comparable stroke and myocardial infarction (MI) rates between conventional CABG and minimally invasive approaches verifying its safety [15]. In line with previous evidence [8,10,16], our study reports no cases of perioperative stroke, no cases of new-onset dialysis, and only two cases (0.64%) of perioperative MI. In our study, we present seven cases (2.3%) of newly manifested AF, which is favorable compared to previously reported rates [8,16]. We report no need for intraoperative blood transfusion in our cohort and a short duration of ICU (1.0, 0–1.0 days) and in-hospital stay (8.7 ± 5.5 days), which is comparable to previously published data [8,9] reporting a significantly lower proportion of transfused patients and a shorter ICU and in-hospital stay amongst MIDCAB patients [16,17,18]. The conversion rate to sternotomy (3 cases, 1%), as well as the conversion to cardiopulmonary bypass (3 cases, 1%), was low and comparable to the evidence in the literature [10,12]. Four patients (1.3%) had to be re-explored for bleeding, a rate which is in line with previously published data [7,10,19]. 

The literature contains extensive debate about the long-term outcomes of MIDCAB compared to other minimally invasive coronary revascularization methods, such as percutaneous coronary intervention (PCI). For patients with multivessel coronary artery disease, evidence suggests that coronary artery bypass grafting offers more favorable outcomes than both medical therapy and PCI. Specifically, medical therapy has been associated with a higher occurrence of subsequent myocardial infarctions, increased need for additional revascularizations, and a higher incidence of cardiac death. Meanwhile, PCI is linked to a greater incidence of myocardial infarctions, an increased requirement for further revascularization, and a 1.46-fold increased risk of combined adverse events compared to CABG [20].

Several studies have shown similar rates of death, myocardial infarction, and stroke amongst PCI patients compared to MIDCAB, albeit with a notably higher rate of repeat revascularization in PCI patients [21,22,23]. A study by Merkle et al. has shown that although MIDCAB operation is linked to a longer ICU and hospital stay, it is associated with notably reduced occurrences of repeat revascularization and lower mortality rates compared to PCI [24].

Recent meta-analyses have highlighted the long-term advantages of MIDCAB over percutaneous coronary intervention. Specifically, studies have indicated that MIDCAB patients experience lower all-cause mortality and reduced rates of repeat revascularization compared to those undergoing PCI [25]. These findings align with earlier meta-analyses demonstrating that MIDCAB not only minimizes the need for subsequent interventions but also achieves comparable mortality rates and incidences of major adverse cardiac and cerebrovascular events to those of drug-eluting stents [26]. Additionally, when comparing minimally invasive left internal thoracic artery bypass to percutaneous transluminal coronary artery stenting for isolated left anterior descending artery lesions, MIDCAB has been shown to lead to fewer mid-term complications, further validating its efficacy and safety [27].

Research has extensively compared the safety and efficacy of the MIDCAB approach with both standard on-pump CABG and sternotomy off-pump CABG (OPCAB) [28]. Notably, a case-matched study by Lapierre et al. demonstrated that MIDCAB led to shorter hospital stays and quicker postoperative recovery compared to OPCAB [29]. This approach is also associated with several other benefits, including lower rates of blood transfusions and wound infections, due to the preservation of sternum integrity [30]. Although the study of Raja et al. further supported the safety of MIDCAB on a mean follow-up of 12.95 ± 0.47 years, it failed to prove its superiority compared to standard CABG [5].

As previously mentioned, our center has recently begun using the new Multistation^TM^ retractor (LSI Solutions, USA) in patients undergoing minimally invasive coronary revascularization. This innovative tool enhances surgical exposure and access, facilitating the harvesting of both the left and right internal mammary arteries (LIMA and RIMA) and allowing for complex multivessel revascularizations through smaller incisions. The Retro-Sterno^TM^ paddle, which is placed through a small 1.5 cm subxyphoidal incision, provides easier access to the RIMA through the left anterolateral thoracotomy, allowing its harvesting both under direct vision or endoscopically. 

Current evidence on the MIDCAB approach is somewhat limited due to a scarcity of large prospective studies and randomized controlled trials, as well as variations in the definitions of techniques and complications, coupled with suboptimal clinical follow-up protocols [31]. Further studies are needed to better investigate the postoperative results of MIDCAB surgery and its benefits compared to other coronary revascularization approaches. 

Due to the demanding learning curve associated with the MIDCAB approach [32,33], it is important to note that maintaining a high quality of the MIDCAB procedure is achievable when it is carried out in high-volume centers, enabling surgeons to effectively sustain their skills [12,32,34]. 

### Limitations

This study presents limitations mainly related to its retrospective, observational, and single-center design. 

The population of the present study comprises patients with diverse disease severity, ranging from single to multivessel coronary disease while also including mostly elective as well as a few emergent cases, which may affect the long-term survival and complication rates. It should also be mentioned that the patient cohort of the present study included a selected group of relatively low-risk patients and favorable baseline patient characteristics, thus the reported results should not be generalized to all with coronary artery disease. Moreover, our cohort presents the entire experience of our center also including the first cases. Taking into consideration the initial learning curve associated with the implementation of every new interventional technique may have impacted our results.

## 5. Conclusions

MIDCAB offers a viable and effective option for surgical myocardial revascularization with favorable long-term outcomes and minimal perioperative complications, particularly in low-risk patients. Our findings support the efficacy and safety of MIDCAB, suggesting its broader adoption in suitable candidates within high-volume centers. Further research is needed to validate these findings through prospective, multi-center trials to overcome limitations associated with retrospective analyses.

## Figures and Tables

**Table 1:** Baseline characteristics.

Parameter	n (%)
age	63.3 ± 10.9
female gender	95 (30.6%)
CAD1	197 (63.5%)
CAD2	71 (22.9%)
CAD3	42 (13.5%)
acute STEMI	2 (0.6%)
acute NSTEMI	15 (4.8%)
previous PCI	73 (23.5%)
active smoker	93 (30%)
IDDM	9 (2.9%)
NIDDM	48 (15.5%)
hyperlipoproteinemia	160 (51.6%)
arterial hypertension	280 (90.3%)
kidney function impairment	25 (8.1%)
terminal KI with dialysis	3 (1%)
LVEF, %	57.1 ± 6.5
EuroSCORE II, %	0.9 (0.7–1.2)

CAD—coronary artery disease, STEMI—ST-elevation myocardial infarction, NSTEMI—non-ST-elevation myocardial infarction, IDDM—insulin-dependent diabetes mellitus, KI—kidney injury, LVEF—left ventricular ejection fraction, and NIDDM—non-insulin-dependent diabetes mellitus.

**Table 2:** Intraoperative data.

Parameter	n (%)
urgency	
elective	236 (76.1%)
urgency	61 (19.7%)
emergent	13 (4.2%)
MIDCAB (LIMA-LAD)	310 (100%)
operating time, min	129.7 ± 35.3
conversion to sternotomy	3 (1%)
conversion to CPB	3 (1%)
blood transfusion, Units	0.0 (0.0–2.0)
blood transfusion	87(28.0%)

CPB—cardiopulmonary bypass, LAD—left anterior descending, LIMA—left internal mammary artery, and MIDCAB—minimally invasive direct coronary artery bypass.

**Table 3:** Postoperative data.

Parameter	n (%)
in-hospital stay, days	8.7 ± 5.5
ICU stay, days	1.0 (0–1.0)
new onset AF	7 (2.3%)
max CK	577.5 ± 430.6
max CK MB	35.5 ± 20.7
max Troponin	28.0 (21.7–37.3)
perioperative myocardial infarction	2 (0.64%)
stroke	0
new onset dialysis	0
postoperative angiography	2 (6.4%)
postoperative LAD intervention, graft occlusion	1 (0.3%)
re-exploration for bleeding	4 (1.3%)
blood transfusion in the ICU, Units	0.0 (0–4.0)

AF—atrial fibrillation, CK—creatine kinase, ICU—intensive care unit, and LAD—left descending artery.

**Table 4:** Follow-up data.

Parameter	n (%)
Follow-up time, years	16.3 ± 6.3
in-hospital mortality	2 (0.64%)
6-months mortality	3 (0.97%)
1-year mortality	3 (0.97%)
5-year mortality	1.3%
10-year mortality	5.7%
Death at follow-up	31 (10%)
cardiac cause of death	6 (1.9%)
neurological cause of death	7 (2.3%)
oncological cause of death	8 (2.6%)
other cause of death	10 (3.2%)
coronary angiography	63 (20.3%)
bypass closure	5 (1.6%)

## Data Availability

The data are available from the corresponding author upon reasonable request.
